# Erratum to: Less is More: Removing a Modality of an Expected Olfactory‐Visual Stimulation Enhances Brain Activation

**DOI:** 10.1002/hbm.26023

**Published:** 2022-07-22

**Authors:** Doris Schicker, Sonja Blankenagel, Claus Zimmer, Hans Hauner, Jessica Freiherr

**Affiliations:** ^1^ Sensory Analytics & Technologies Fraunhofer Institute for Process Engineering and Packaging IVV Freising Germany; ^2^ Department of Psychiatry and Psychotherapy Friedrich‐Alexander‐Universität Erlangen‐Nürnberg Erlangen Germany; ^3^ Plant Breeding, TUM School of Life Sciences Technical University of Munich Freising Germany; ^4^ Department of Diagnostic and Interventional Neuroradiology, School of Medicine, Klinikum rechts der Isar Technical University of Munich Munich Germany; ^5^ ZIEL ‐ Institute for Food & Health Technical University of Munich Freising Germany; ^6^ Institute for Nutritional Medicine, Else Kröner‐Fresenius‐Centre for Nutritional Medicine, School of Medicine Technical University of Munich Munich Germany

The authors would like to correct the Acknowledgements (Schicker et al., 2022) to the following updated Acknowledgements section:

We are grateful to Christoph Hofstetter and Andreas Dunkel for the development of the food preference questionnaire as well as the shared data collection using the questionnaire and for the supply of the biomimetic aroma recombinates. We would like to thank the Neuroradiology team of Klinikum Rechts der Isar for their technical support during data acquisition. We also would like to thank Kathrin Koch und Tim Rohe for fruitful discussion of the results of the study. Finally we would like to thank Alyssa Torske for language revision of the manuscript. This work was supported by a grant of the German Ministry for Education and Research (BMBF, grant no. 01EA1409A), the German Academic Scholarship Foundation, as well as the Initiative Campus of the Senses, a project with financial support of the Bavarian Ministry of Economic Affairs, Regional Development and Energy (StMWi) and the Fraunhofer Society. The present work was performed in partial fulfillment of the requirements for obtaining the degree “Dr. rer. biol. hum.” at Friedrich‐Alexander‐Universität Erlangen‐Nürnberg. Open access funding enabled and organized by Projekt DEAL.

## Data Availability

Data sharing is not applicable to this article as no new data were created or analyzed in this study.
